# Special Issue on Skin Cancers of the Head and Neck

**DOI:** 10.3390/cancers18010030

**Published:** 2025-12-21

**Authors:** Kaiwen Chen, Shaun A. Nguyen, Kaitlyn A. Roberts, Warren B. Chun, Cherie-Ann O. Nathan

**Affiliations:** 1 Department of Otolaryngology–Head and Neck Surgery, Medical University of South Carolina, Charleston, SC 29425, USA; 2 Department of Otolaryngology–Head and Neck Surgery, LSU Health Shreveport, Shreveport, LA 71103, USA; cherieann.nathan@lsuhs.edu

In this Special Issue of *Cancers*, we bring together a collection of five studies that capture the rapidly evolving landscape of skin malignancies of the head and neck. These works highlight major developments in genomics, immunotherapy, and the management of skin cancers specifically affecting the head and neck. We hope this Special Issue will inform clinicians and researchers about the current state of the field and inspire future directions.

Skin cancer remains among the most prevalent cancers worldwide, with the incidence rising annually. Melanoma and non-melanoma skin cancers together account for over 90% of all skin cancers [1]. Because ultraviolet radiation is a major risk factor for all types of skin cancer, the head and neck region experiences the highest cumulative ultraviolet exposure and therefore disproportionately bears the disease burden. Rising global incidence, especially in sun-exposed regions, highlights the importance of improved risk assessment tools tailored to head and neck disease [2,3,4]. Despite the availability of risk stratification systems such as those from the American Joint Committee on Cancer (AJCC), the Brigham and Women’s Hospital system (BWH), and the National Comprehensive Cancer Network guidelines (NCCN), predictive accuracy for metastatic potential remains limited [5,6,7].

In recent years, gene expression profiling (GEP) has emerged as a promising diagnostic tool, offering molecular insights not captured through conventional clinicopathologic methods. By providing molecular data beyond routine histopathology, GEP offers enhanced prognostic accuracy and supports individualized management [8,9]. Two reviews by Durgham and colleagues in this Special Issue evaluate the role of GEP in melanoma and subcutaneous squamous cell carcinoma, both of which commonly arise on the head and neck due to ultraviolet exposure.

Cutaneous melanoma, the fifth most common cancer in the United States, illustrates this advancement toward molecular precision [10]. Although melanoma represents only 1–2% of all skin cancers, it causes the majority of skin cancer-related deaths due to its metastatic potential [11,12,13]. The 31-gene expression profile (31-GEP) and the MelaGenix assays improve prognostic accuracy in ways that are particularly meaningful for head and neck melanoma, which may be more challenging to detect early. In a systematic review and meta-analysis, Durgham et al. demonstrated that 31-GEP class stratifications correlated significantly with five-year and three-year melanoma-specific, recurrence-free, and distant metastasis-free survival rates, supporting the role of molecular tools to guide clinical decision making [9]. Their findings emphasize the importance of molecular risk stratification as a complement to AJCC staging, improving identification of high-risk patients who may benefit from closer surveillance or adjuvant therapy. A review by Durgham et al. found that the 40-gene expression profile (40-GEP) test more accurately predicts time to local recurrence or metastasis, and that more advanced classes were independent risk factors for metastasis. The review also found that class 2B had a higher positive predictive value for metastasis than the AJCC or BWH staging systems. These findings suggest that integrating GEP testing allows for more nuanced risk stratification and the potential for more individualized patient management [14,15,16]. Collectively, these studies mark an important shift toward molecularly guided precision care in head and neck skin oncology.

Cutaneous squamous cell carcinoma (cSCC), the second most common skin cancer, is one of the most frequent malignancies of the head and neck, and genomic profiling helps refine risk predictions [17]. With multiple genes and pathways implicated in the development and progression of cSCC, disease course and patient outcomes vary widely [18,19]. The 40-GEP test categorizes tumors into risk groups that outperform traditional staging systems, which often underpredict aggressive behavior in the head and neck.

Prognosis of cSCC varies widely and is strongly influenced by factors that are especially common in head and neck tumors, including perineural involvement and location near critical structures [20,21,22]. Immunosuppression is particularly important, as sun-exposed areas of the scalp, face, and ears are high-risk sites in these patients [23,24]. In this Special Issue, Hwang and St. John present a comprehensive review entitled *“Cutaneous Squamous Cell Carcinoma in Immunocompromised Patients”*. Their work summarizes current evidence on how immunosuppression from organ transplantation, hematologic malignancies, autoimmune disorders, and chronic infections alters tumor behavior, recurrence rates, and survival [25]. They highlight that existing staging systems, such as AJCC and BWH, may underestimate disease risk in immunocompromised patients and recommend that immune status be integrated into future prognostic models. By outlining mechanisms of tumorigenesis, the impact of immunosuppressive drugs, and preventive strategies, they provide a clear framework for management of this high-risk population [26].

Immunotherapy has reshaped treatment pathways for high-risk melanoma and other cutaneous cancers of the head and neck. Checkpoint inhibitors have been shown to significantly improve both overall survival and progression-free survival in metastatic melanoma [27,28,29]. Neoadjuvant immunotherapy improves pathologic response rates and may reduce surgical morbidity without increasing complications [30]. Mansour et al. reviewed these developments and found that agents such as pembrolizumab, ipilimumab, and nivolumab improved event-free survival, recurrence-free survival, and pathologic response. Although some therapy combinations resulted in increased toxicity, surgical complications did not rise [31]. Recent clinical trials have demonstrated that combination neoadjuvant therapy achieves higher rates of complete or near-complete pathologic response compared with standard-of-care adjuvant therapy [32,33]. As dosing regimens become more tailored and newer agents are introduced, the future of neoadjuvant therapy shows great promise as safety and tolerability continue to improve [34,35].

Merkel cell carcinoma (MCC) is a rare but aggressive cancer frequently arising in chronically sun-exposed head and neck skin, making it an important focus of emerging immunotherapy trials. With disease-related mortality rates between 25 and 50%, current clinical trials are underway to evaluate new immunotherapy regimens, direct tumor injections, and alternative radiation dosing [36,37]. Patel et al. have highlighted these emerging directions in their review, *“Current Trends in Clinical Trials for Merkel Cell Carcinoma (MCC).”* [38]. Multiple studies have shown that PD-1 and PD-L1 checkpoint inhibitors are efficacious and safe options even in advanced MCC, and ongoing trials are exploring their use in earlier stages and combination regimens. Novel molecular targets such as the inosine monophosphate dehydrogenase 2 (IMPDH2) and nicotinamide N-methyltransferase (NNMT) are also under investigation, offering new directions for therapeutic discovery [39,40,41].

Together, these manuscripts demonstrate advances in molecular diagnostics, immunotherapy, and staging approaches that are particularly relevant for head and neck cutaneous oncology. This Special Issue aims to guide clinicians and researchers towards more precise and individualized care for patients with skin cancers of the head and neck.
